# Advances in Next-Generation Coronavirus Vaccines in Response to Future Virus Evolution

**DOI:** 10.3390/vaccines10122035

**Published:** 2022-11-29

**Authors:** Lili Li, Yangyang Wei, Han Yang, Junyu Yan, Xin Li, Ziqian Li, Yuxiu Zhao, Hongyang Liang, Hui Wang

**Affiliations:** Beijing Institute of Biological Products Company Limited, Beijing 100176, China

**Keywords:** COVID-19, pandemic, variants, vaccine, immune response, pan-coronavirus vaccines, virus evolution

## Abstract

Coronavirus disease (COVID-19), caused by severe acute respiratory syndrome coronavirus 2 (SARS-CoV-2), has spread to more than 230 countries and territories worldwide since its outbreak in late 2019. In less than three years, infection by SARS-CoV-2 has resulted in over 600 million cases of COVID-19 and over 6.4 million deaths. Vaccines have been developed with unimaginable speed, and 11 have already been approved by the World Health Organization and given Emergency Use Listing. The administration of several first-generation SARS-CoV-2 vaccines has successfully decelerated the spread of COVID-19 but not stopped it completely. In the ongoing fight against viruses, genetic mutations frequently occur in the viral genome, resulting in a decrease in vaccine-induced antibody neutralization and widespread breakthrough infection. Facing the evolution and uncertainty of SARS-CoV-2 in the future, and the possibility of the spillover of other coronaviruses to humans, the need for vaccines with a broad spectrum of antiviral variants against multiple coronaviruses is recognized. It is imperative to develop a universal coronavirus or pan-coronavirus vaccine or drug to combat the ongoing COVID-19 pandemic as well as to prevent the next coronavirus pandemic. In this review, in addition to summarizing the protective effect of approved vaccines, we systematically summarize current work on the development of vaccines aimed at suppressing multiple SARS-CoV-2 variants of concern as well as multiple coronaviruses.

## 1. Introduction

Coronaviruses are a large group of enveloped single-stranded RNA viruses that can infect a variety of vertebrates [1]; the four recognized types are alpha (α), beta (β), gamma (γ), and delta (δ). In the last two decades, three β-coronavirus strains with high pathogenicity and lethality have emerged as a result of zoonotic transmission. These include severe acute respiratory syndrome coronavirus (SARS-CoV-1), which emerged in China in 2002; Middle East respiratory syndrome coronavirus (MERS-CoV), which emerged in Saudi Arabia and South Korea in 2012; and severe acute respiratory syndrome coronavirus 2 (SARS-CoV-2), which was first identified in Wuhan, China in late 2019 and is the cause of coronavirus disease 2019 (COVID-19) [2,3]. SARS-CoV-2 was declared a pandemic by the World Health Organization (WHO) on 11 March 2020, and it has spread to almost all countries [4]. Currently, 11 COVID-19 vaccines have been assessed to be safe and effective by WHO (Table 1) [5,6,7,8,9,10,11]. As of 12 September 2022, 138 vaccines are being clinically tested [12]. Vaccines developed using different technology platforms have been widely used to immunize populations worldwide since December 2020 [13]. The COVID-19 vaccines are highly protective against severe illness, hospitalization, and death, although the efficacy of these vaccines declines 6 months after full vaccination, and they cannot prevent breakthrough infections [14]. The vaccines appear to be considerably less protective against the Omicron variant, and the development of new antibody therapies has not progressed in line with the rate at which the virus is mutating the neutralizing epitopes [15,16,17]. There is no drug that is completely effective, and symptomatic treatments are widely followed in clinical therapeutics against SARS-CoV-2 [18]. Additional challenges loom for the next phase in building a future-proof and durable immune repertoire to protect against oncoming viral variants.

## 2. New Situation Caused by SARS-CoV-2 Evolution

**Figure 1:**
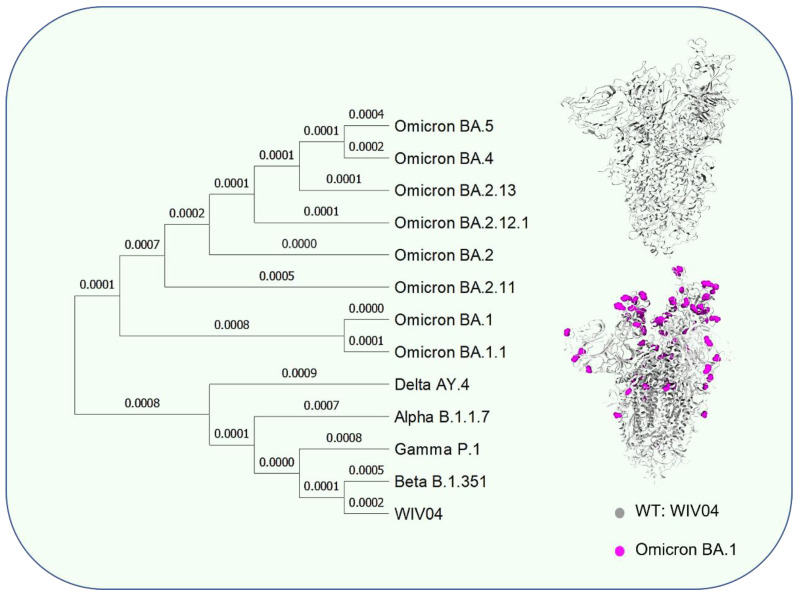
Phylogenetic tree using neighbor-joining method and schematic diagram of mutation sites. Phylogenetic tree was generated by MEGAsoftware (version 11.0.13). The genomes of CoV variants were retrieved from GISAID (https://www.gisaid.org/, accessed on 2 November 2022). WIV04: EPI_ISL_402124; Alpha: EPI_ISL_674612; Beta: EPI_ISL_940877; Gamma: EPI_ISL_2777382; Delta: EPI_ISL_1758376; Omicron BA.1: EPI_ISL_6795848; Omicron BA.1.1: EPI_ISL_8724963; Omicron BA.2: EPI_ISL_8135710; Omicron BA.4: EPI_ISL_11542550; Omicron BA.5: EPI_ISL_11542604; Omicron BA.2.12.1: EPI_ISL_11368598; Omicron BA.2.11: EPI_ISL_9862290; Omicron BA.2.13: EPI_ISL_9512109.

SARS-CoV-2 has been constantly mutating over the course of the pandemic for the last three years [19,20,21]. Naturally occurring genetic variation during replication is more frequent in RNA viruses than in DNA viruses, despite the presence of exonucleases [22,23]. In addition, directed mutation pressure exerted by the host antiviral immune defense system accelerates genetic variation [24,25]. During late 2020, the emergence of variants that posed an increased risk to global public health prompted WHO to conduct more detailed characterization of specific variants of interest (VOI) and variants of concern (VOC), to monitor the variations in the virus and release information on virus variations while working with scientists around the world to deal with the change of the virus. All reported VOCs include five mutant strains: alpha (B.1.1.7), beta (B.1.351), gamma (P.1), delta (B.1.617.2), and omicron (B.1.1.529) [26]. Although these VOCs originated in different countries, a common feature among them is the increase in the number of mutation sites, with the virus becoming more transmissible [27,28,29]. Omicron B.1.1.529 was listed as a VOC on 26 November 2021; it has 36 deletions and mutations in the spike protein compared with the wild type. Compared with the mean reproduction number (R0) of the original strain (2.79), the delta variant has an R0 of 5.08 [30] and the omicron variant has an R0 of more than 8 [30]. During 2022 when the omicron variant was widespread, multiple mutations occurred, and multiple branches evolved (Figure 1).

**Figure 2:**
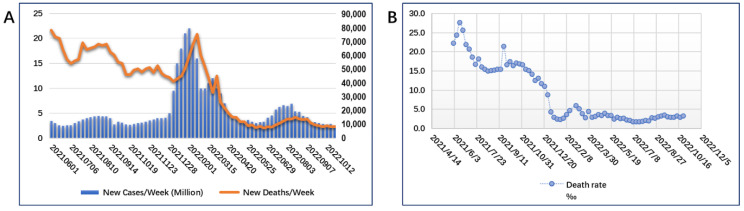
Infections and deaths since June 2021 to October 2022. Infections of SARS-CoV-2 sharply increased and deaths rate has been gradually declining in the early 2022 since Omicron variant became the predominant epidemic variant. (**A**), The weekly number of new cases and new deaths worldwide (from June 2021 to October 2022) released by the WHO. (**B**) Global death rates due to COVID-19 infection (ratio of deaths to infections) from June 2021 to October 2022.

In late 2021, the omicron variant became the predominant epidemic variant. Unlike previous VOCs, the prevalence of omicron caused some changes in the epidemiological parameters of the virus. As shown in Figure 2A, the number of weekly infections sharply increased in early 2022, from 5 million (June 2021 to December 2021) to more than 15 million, indicating that the infection and transmission capacities of the omicron variant are significantly higher than those of the previous VOCs. In contrast, the number of deaths (the orange curve: Figure 2A) was gradually declining during the period when omicron became a major prevalent VOC (December 2021 to January 2022); the death rate from COVID-19 infection dropped from over 15% to less than 5 (Figure 2B). Although vaccination did not alter breakthrough infections, it had a major effect in reducing severe illness and mortality. A pathogenicity study of omicron infection showed that pathogenicity was significantly attenuated in both upper and lower respiratory tracts [31].

Many studies have shown that inactivated vaccine, viral vector vaccine, and mRNA vaccine based on the genome of the original virus strain have shown significantly lower protection efficiency against the omicron variant [32,33]. In comparing the previous VOC with omicron, administering COVID-19 vaccines provided limited protection against SARS-CoV-2 infection but high protection against severe/critical illness and COVID-19-related deaths in all age groups [34,35,36].

## 3. Next-Generation Vaccine Design against Mutated Viruses

**Figure 3:**
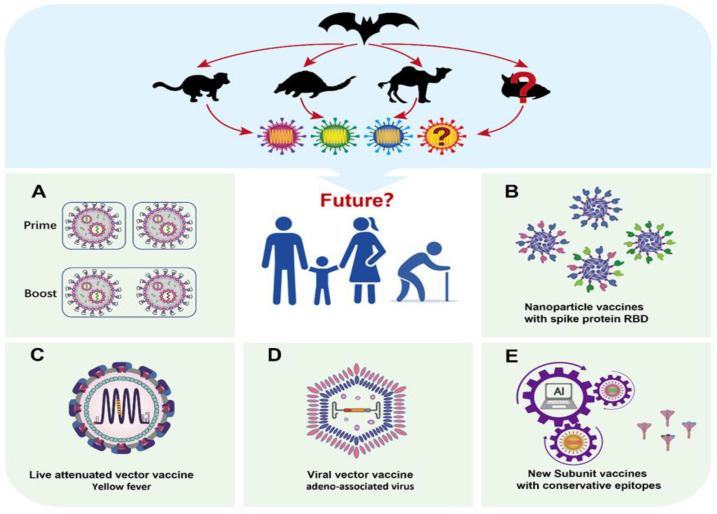
Applied platform for next-generation anti-coronavirus R&D. Booster immunization with approved vaccines (**A**), Vaccines with more complex sources of spike protein RBD (**B**), Live attenuated viral vaccine delivered SARS-CoV-2 antigen (**C**), Subunit vaccines with T-cell epitopes (**D**). More cutting-edge vaccine design using AI technology (**E**) can be used for the prevention of infectious diseases.

Numerous studies have shown that the mutations in the omicron variant allow it to evade the protection conferred by vaccines and therapeutic antibodies, which is a concern for people worldwide [37,38,39,40,41]. In light of the COVID-19 pandemic and the unpredictable nature of the virus, there is a growing belief that the virus may not be wiped out completely but rather will coexist with humans for a long time [42,43]. In addition to closely monitoring virus transmission and developing therapeutic drugs, a more profound and the most cost-effective, long-term solution is to develop safe and effective next-generation pan-coronavirus vaccines. Available data indicate that the problems with the approved vaccines are the short duration of the high levels of protective antibodies induced and the insufficient cross-protection against mutated viruses [16]. A strategy to overcome the problems is to boost with homologous and heterologous vaccines in order to increase the titer of cross-reactive antibodies and prolong the duration of protection, such as regular annual booster shots of available vaccines, as is practiced for influenza [44,45]. Another strategy is to include more key epitopes of SARS-CoV-2 or even epitopes of other coronaviruses in the next-generation vaccine design in order to increase the chance of generating more cross-protective antibodies to deal with new mutations of the virus. Furthermore, some studies have used approved vaccine (such as other vaccine strains) as delivery vectors to exert their strong immune activation to increase vaccine immunogenicity. The persistent expression of coronavirus antigens by infection with a delivery vector has also been tested. Moreover, several technologies, including bioinformatic computing and prediction functions [46,47], reverse vaccinology and machine learning [48,49], as well as nanoparticle vectors, viral vectors other than adenoviruses, and chimeric spike mRNA vaccines, have been used, with some positive results. The following sections provide a detailed review of these next-generation coronavirus vaccine development strategies (Table 1 and Figure 3).

## 4. Strategy One: Booster Immunization with Approved Vaccines

Using an approved vaccine as a booster is certainly a quick and cost-effective option to deal with the mutation of novel coronavirus, although data from multiple clinical trials have shown that the titers of neutralizing antibodies decrease 6 months after immunization with any of the approved SARS-CoV-2 vaccines [33,50,51,52,53]. Results of booster doses 3 and 4 using homologous or heterologous approved vaccines have shown that 2 doses of primary immunization provided limited protection, and the third booster, whether homologous or heterologous, could effectively increase the levels of neutralizing antibodies against the omicron strain, but it decreased significantly after 10 weeks [32,54,55]. The data suggest that booster with a heterologous vaccine may be better than a homologous vaccine [56]. The fourth booster did not significantly prolong the duration of vaccine efficacy compared with the third booster, except for slightly increasing the antibody levels [57]. In addition, vaccines with omicron antigen, which work on the same platform as the approved vaccine, are also being tested in clinical trials [58]. To maintain protection against omicron, regulatory agencies have approved a third dose for healthy vaccinees who are at a risk of the clinical consequences of the postvaccination drop in immunity [59]. There is no doubt that booster immunization with approved vaccines can provide protection against new mutated viruses for a relatively limited time.

## 5. Strategy Two: Vaccines with More Complex Sources of Spike Protein RBD

SARS-CoV-2 relies on its obligate receptor, angiotensin-converting enzyme 2 (ACE2), to enter cells [60,61,62].The spike protein is a trimeric glycoprotein that comprises two domains: the S1 domain, which contains the RBD, and the S2 domain, which is responsible for membrane fusion [63,64,65,66,67]. The spike protein RBD has become a prime focus in the research and development of pan-coronaviruses because it is the main receptor recognition site, the binding site for neutralizing antibodies, and the site where mutations occur most frequently and gradually increase with virus evolution [68,69,70,71,72,73,74]. The expressed soluble spike protein is the main antigen in all approved vaccines, except inactivated vaccines [9,75,76]. However, previous studies have shown that particle antigens are more effective than free antigens. The repetitive array of the particle surface component allows for robust B cell activation that facilitates memory B cell expansion and the generation of long-lived plasma cells [77,78]. Based on this principle, self-assembling nanoparticles are being used in vaccine research because they provide a robust platform to investigate the concept of particulate vaccines such as virus-like particles (VLPs). Furthermore, self-assembling nanoparticles can improve antigen structure and stability, as well as vaccine-targeted delivery and immunogenicity, all with a good safety profile [79]. Ferritin is a protein that naturally forms nanoparticles composed of 24 identical polypeptides [80,81,82]. Genes encoding immunogenic substances are inserted at the interface of adjacent subunits enabling spontaneous assembly and the generation of eight trimeric viral spikes on the nanoparticle surface [83]. The most advanced ferritin nanoparticle SARS-CoV-2 vaccine, S-trimer-ferritin nanoparticles (SpFN), is in phase I clinical trials conducted by the U.S. Army Medical Research and Development Command (NCT04784767). Preclinical studies demonstrated that SpFN combined with ALFQ, a liposomal adjuvant, can induce potent S-binding and ACE2 inhibiting neutralizing antibody; binding titers and pseudovirus neutralization titers were stably maintained for more than 10 weeks after the final immunization in mice [84]. In another research study, SpFN protected hamsters against alpha and beta coronavirus variants [85]. In nonhuman primate experiments, the vaccine generated more potent SARS-CoV-2-specific B- and T-cell responses than a soluble antigen vaccine, and it protected Chinese-origin rhesus macaques in high-dose virus challenge experiments. SpFN elicited serum virus-neutralizing activity that was either higher than or equivalent to that of the wild-type virus against four major VOCs (B.1.1.7, B.1.351, P.1, and B.1.617.2) in an authentic virus neutralization assay [86].

In the face of coronavirus spillover from animals to humans and unpredictable changes in the virus, mosaic RBD nanoparticles are being used in vaccine research. Cohen et al. constructed several nanoparticles (mosaic-4a, mosaic-4b, mosaic-8, and homotyic SARS-2) that simultaneously displayed RBDs from human and animal coronaviruses [87]. Mice experiments showed that the mosaic RBD nanoparticle vaccine produced neutralizing antibodies and cross protection against multiple coronaviruses, such as bat WIV1 and SHC014 strains, and the antibody level was equal to or greater than that of the single RBD nanoparticle [88]. In a further study, when mosaic-8 nanoparticles were used to immunize mice and nonhuman primates, the animals were protected against SARS-CoV and SARS-CoV-2, including Omicron [89].

In addition to recombinant nanoparticle vaccines, mixing mRNA vaccines with different virus variants Spike proteins are also being developed, and these bivalent mRNA vaccine include a component of the original virus strain to provide broad protection against COVID-19 and a component of the omicron variant to provide better protection against COVID-19 caused by the omicron variant. [90]. In addition, chimeric spike mRNA vaccines were used in one study; these are LNP vaccines that are consistent with approved mRNA vaccines, except that these vaccines express chimeric spikes that contain admixtures of different RBD from SARS-CoV, SARS-CoV-2, SARS-CoV-2 B.1.351, and bat CoV (Bt-CoV) RsSHC014;d a heterologous Bt-CoV WIV-1, N-terminal domain; and S2 modular domains from zoonotic, epidemic, and pandemic coronaviruses. In vivo, the vaccine induced neutralizing antibodies comparable with that of a monovalent vaccine, and old mice were completely protected from bat-derived BT-CoV RsSHC014 and SARS-CoV-2. An advantage of the chimeric spike vaccines is the clear breadth of protection against multiclade sarbecoviruses (SARS-CoV, Bt-CoV RsSHC014, Bt-CoV WIV-1) and SARS-CoV-2 variants (B.1.351) compared with that of monovalent SARS-CoV-2 vaccines [91].

Recombinant nanoparticle vaccines and chimeric spike mRNA vaccines allow for a denser concentration of antigen material and can carry multiple coronavirus antigens, providing additional opportunities for cross protection against mutations of SARS-CoV-2 and unpredictable coronavirus spillovers in the future. We also know that too many antigenic substances not associated with epidemic infections are added to vaccines, which are not recommended by regulators. Perhaps the major challenge for such a vaccine is to predict mutations in the virus, as with influenza, before developing and producing a vaccine and conducting more experiments on safety and efficacy.

## 6. Strategy Three: Live Attenuated Viral Vaccine Delivered SARS-CoV-2 Antigen

Attenuated vaccines are widely used against various infectious diseases such as smallpox [92,93], Varicella [94,95], Japanese encephalitis [96,97], and yellow fever [98,99]. This type of vaccine is highly immunogenic [100,101], safe based on its history of use [102,103]. Live attenuated vaccine viruses are used for the delivery of antigenic substances and are also widely researched for the treatment of tumors and other diseases [104,105,106,107]. Using this principle, an antigen of SARS-CoV-2 or other coronaviruses is inserted into the attenuated viral genome providing another technology platform for pan-coronavirus vaccine development.

YF17D is a small positive-sense single-stranded RNA live-attenuated virus vaccine for yellow fever virus (YFV) that tolerates some insertion of foreign antigens in the viral polyprotein [108]. A single-dose YF17D vaccination can induce lifelong protective immunity against YFV, and it is well- tolerated in humans, with highly rare adverse events [109,110]. In recent years, YF17D has been used in some vaccine research as an alternative viral vector vaccine platform [111,112,113,114]. Oreshkova et al. [115] established a live attenuated vaccine using YF17D as the delivery vector that comprised a spike protein of three different beta-coronaviruses: MERS-CoV, SARS-CoV, and MHV replaced prM and E of YF17D. The vaccine induced the production of corresponding antibodies on day 42 after booster vaccination. Sanchez-Felipe et al. [116] found that a single dose of YF17D-VECtored SARS-CoV-2 vaccine had a good safety profile in hamsters and nonhuman primates, and it could protect animals from viral attack.

Modified vaccinia Ankara (MVA) is another vaccine candidate for SARS-CoV-2 [117] that induces strong, broad, and polyfunctional SARS-CoV-2 S-specific T-cell immunity and high level B-cell immunity with good safety in mice and hamsters [117,118,119,120]. A recombinant MVA expressing full-length SARS-CoV-2 spike (S) protein named MVA-SARS-2-S was developed by Universitatsklinikum Hamburg-Eppendorf in Germany and has completed Phase I clinical trials (NCT04569383). Preclinical test results confirmed that poxvirus MVA has strong immunogenicity, and S protein can be expressed at a high level in animals to induce strong humoral and cellular immune responses. Vaccines stimulate the production of neutralizing antibodies and protect animals from viruses in protective challenge tests [121]. In addition, Flavia Chiuppesi’s team developed vaccines carrying co-expressing SARS-CoV-2 spike and nucleocapsid antigens using MVA viruses [117].

Live attenuated measles virus (MeV) vaccine has been one of the safest and most efficient human vaccines and has been used in children since the 1960s [122,123]. A measles virus (rMeV) vaccine strain as the backbone expressing various forms of the SARS-CoV-2 spike protein and RBD has been used in animal experiments and was demonstrated to induce high levels of antibodies and a strong Th1-biased T-cell response in vivo, while protecting animals against SARS-CoV-2 infection [124].

In addition to the live attenuated virus, Adeno-associated viruses are commonly used as viral vectors for gene therapy characterized by low immunogenicity and can continuously express the delivered gene after infection [125,126]; they have also been used in a novel coronavirus vaccine. Previous studies have also confirmed that AAV vector vaccines have a good safety profile and induce durable and effective humoral and cellular immunity in mice and nonhuman primates [127,128,129]. In a previous study, the S protein-encoding gene of the original SARS-CoV-2 strain was integrated into AAVrh32.33; the neutralizing antibody levels decreased in treated mice for up to a year. The treatment also induced a durable cellular immune response and provided complete protection against SARS-CoV-2 challenge in cynomolgus monkeys [130]. In another study, AAV6 virus with the spike protein-encoding gene of the original strain was compared with an approved inactivated vaccine in animals and showed that a single dose produced a higher titer and longer lasting IgG antibody [131].

The live virus vector vaccine takes advantage of the characteristics of the virus vector to induce not only a high humoral immune response but also a cellular immune response, which can prolong the protective effect of the vaccine in response to the mutation of the coronavirus.

## 7. Strategy Four: Subunit Vaccines with T-Cell Epitopes

In classical immunology, serum antibody output would be attenuated in the absence of sustained antigen stimulation, but B and T memory cells would be primed for a rapid, protective response upon repeated antigen encounters [132]. Preclinical studies of SARS-CoV have shown a protective role of T cells in the host defense [133]. Together, the induction of a T-cell immune response and the generation of memory T and B lymphocyte immunity have become an important focus of vaccine design against SARS-CoV-2 mutations [134,135,136].

A recombinant subunit vaccine, UB-612, has been developed by expressing the spike 1 (S1)-RBD fused with a single-chain Fc protein (S1-RBD-sFc), 5 Th cell, and CTL epitope peptides from the nucleocapsid (N), membrane (M), and S2 proteins of sarbecoviruses; and an extrinsic HLA class II epitope (UBITh1a) modified from a measles virus fusion (MVF) protein that would serve as a catalyst for T-cell activation [137]. The virus was used in a clinical trial of a multitarget long-acting vaccine against multiple SARS-CoV-2 variants conducted by Chang Yi Wang et al. in Taiwan, China. In completed clinical trials (ClinicalTrials.gov: NCT04545749, NCT04773067, and NCT04967742), the vaccine had a favorable safety profile both as a primary and as a third-dose booster. Compared with a panel of human convalescent sera (HCS) collected approximately 1 month after COVID-19 onset in hospitalized cases, the post-booster neutralizing antibody levels (GMFIs) were about 40-fold higher. The most striking findings were a long half-life of 187 days for the neutralizing antibody response, a sustained T-cell response, and a strong booster-recalled memory immunity with high cross-reactive neutralizing titers against the delta and omicron VOCs [138].

Subunit vaccines with conserved T cell epitopes may cause the body to generate cellular immune response to assist the production of antibodies which are high titer and durable.

## 8. Strategy Five: More Cutting-Edge Vaccine Design Using AI Technology

In addition to clinical studies for SARS-CoV-2, preclinical research has been devoted to the search for T and B cell epitopes in the coronavirus. Through phylogenetic and sequence ratio analyses of coronaviruses, Li et al. [139] investigated the conserved epitopes found in all known human coronaviruses (HCoVs). They then developed a novel antigen based on a genetic algorithm using a mosaic strategy and a broad coverage of CTL epitopes of known HCoVs [139]. Swayam Prakash’s team compared the SARS-CoV-2-Wuhan-Hu-1 (MN908947.3) protein sequence with SARS-CoV- and MERS-CoV-specific protein sequences obtained from human, bat, pangolin, civet, and camel. A total of 15 B cell epitopes, 27 potential human CD8+ T cell epitopes, and 16 potential CD4+ T cell epitopes were highly conserved among 6 strains of coronaviruses previously reported to infect humans. In addition, over 81,000 strains of SARS-CoV-2 that currently circulate in 190 countries on 6 continents were predicted using immune-informatics and sequence alignment. Mice immunized with a mixture of peptides and adjuvants produced IgG against a variety of viruses, some of which were specific for the epitope peptide [140]. Moreover, Ong et al. [48] applied reverse vaccinology tools developed using machine learning to predict COVID-19 vaccine candidates. They proposed that an Sp/Nsp cocktail vaccine containing structural protein and nonstructural protein would stimulate effective complementary immune responses.

AI computing can obtain the genetic information of coronavirus from various sources through big data analysis and predict the evolutionary trend of the virus, which is likely to be an important aid in the design of future vaccines.

## 9. Conclusions

During the three years of the coronavirus epidemic, viruses evolve as they spread. Several data sources have confirmed that infection with the omicron variant reduces the effectiveness of approved vaccines and the neutralizing power of antibodies. It is not impossible to develop new variant strain vaccines to catch up with virus mutation, but from a global vaccine equity perspective, such an effort will not meet the global demand. It has been confirmed that SARS-1, MARS, and SARS-2 are caused by the spillover of animal viruses. For the unknown virus infection in the future, scientists from different countries are using different strategies to carry out research work.

Because of the prevalence of the virus and the danger of the early virus, many countries around the world have developed the COVID-19 vaccine virus at the fastest speed and in the shortest time, which has been approved for use by WHO. Up to now, more than 68.4% of the world’s population has received at least one dose of COVID-19 vaccine, which also means that the next generation of vaccine is closer to the booster vaccine. The booster immunization of vaccine can provide stronger immune protection in a shorter time. Clinical trials also confirmed that heterologous immunization can produce higher neutralizing antibody titer, although the neutralizing antibody level will decrease with the variation of the virus as a whole [141]. The use of heterologous vaccines consistent with antigenic substances and epidemic strains for strengthening may provide better protection for high-risk people.

In the race against viral mutations, more persistent and high-level humoral immune responses against the spike protein and RBD, stronger vaccine immunogenicity, broader pan-coronavirus cross protection, and greater T-cell immune responses have become the consensus for the development of next-generation coronavirus vaccines.

Research on the next-generation vaccine is in progress, and some positive results have been obtained. Future studies should focus on the following aspects: the immune mechanism and protective effect of the vaccine and whether the vaccine can have the expected effect and good safety. The use of new technologies has led to more ideas for vaccine development, but it will require time to prove whether such vaccines are safe and effective. In addition, there are still many unknowns to be explored in the immunological study of coronavirus infection. Immune imprinting caused by vaccination and earlier VOC infection vary in different individuals, and immune responses to new variants of the virus and next-generation vaccines are unknown. Although some vaccines against the mutant strains have already been tested in clinical trials and safety data have been obtained, scientists around the world still need to explore and study the unpredictable evolution of the virus in the future.

## Figures and Tables

**Table 1:** Review of next-generation coronavirus vaccines in development.

Type of Vaccine	Vaccine	Sponsor	Properties	Status
**Booster immunization with approved vaccines**	Comirnaty	Pfizer-BioNTechr	Boost used a bivalent vaccine using the same technology of approved vaccine	approved by FDA
	Spikevax	Moderna		approved by FDA
**Vaccines with more complex sources of spike protein RBD**	SpFN	U.S. Army Medical Research and Development Command	Ferritin nanoparticle with prefusion- stabilized spike antigens from the Wuhan strain of SARS- CoV-2	Phase 1
	Mosaic-4a/4b/8	California Institute of Technology	Ferritin nanoparticle with prefusion- spike antigens from different Coronavirus	Preclinical
	Chimera 1 /4	University of North Carolina at Chapel Hill	Chimeric spike mRNA vaccines with epitops come from differet Coronavirus	Preclinical
**Live attenuated viral vaccine delivered SARS-CoV-2 antigen**	YF-S0	KU Leuven Department of Microbiology, Belgium.	live-attenuated yellow fever 17D (YF17D) vaccine as a vector to express a noncleavable prefusion form of the SARS-CoV-2 spike antigen.	Preclinical
	MVA-SARS-2-S	Universitätsklinikum Hamburg-Eppendorf	recombinant MVA expressing full-length SARS-CoV-2 spike (S) protein	Phase 1
	rMeV-preS	The Ohio State University	measles virus (rMeV) vaccine strain as the backbone expressing SARS-CoV-2 spike (S) protein and its receptor binding domain (RBD)	Preclinical
**Subunit vaccines with T-cell epitopes**	UB-612	United Biomedical Inc., Asia	recombinant subunit vaccine expressing the spike 1 (S1)-RBD fused with a single-chain Fc protein (S1-RBD-sFc), 5 Th cell, and CTL epitope peptides from the nucleocapsid (N), membrane (M), and S2 proteins of sarbecoviruses	Phase 1

## Data Availability

Not applicable.
